# Attenuating effect of *Acorus calamus *extract in chronic constriction injury induced neuropathic pain in rats: an evidence of anti-oxidative, anti-inflammatory, neuroprotective and calcium inhibitory effects

**DOI:** 10.1186/1472-6882-11-24

**Published:** 2011-03-22

**Authors:** Arunachalam Muthuraman, Nirmal Singh

**Affiliations:** 1 Department of pharmaceutical sciences and drug research, Punjabi university, Patiala-147002, India

## Abstract

**Background:**

*Acorus calamus *(family: *Araceae*), is an indigenous plant, traditionally it is used as an ingredient of various cocktail preparations and for the management of severe inflammatory disorders in Indian system of medicine. Present study investigated the attenuating role of *Acorus calamus *plant extract in chronic constriction injury (CCI) of sciatic nerve induced peripheral neuropathy in rats.

**Methods:**

Hot plate, plantar, Randall Selitto, Von Frey Hair, pin prick, acetone drop, photoactometer and rota-rod tests were performed to assess degree of thermal, radiant, mechanical, chemical sensation, spontaneous motor activity and motor co-ordination changes respectively, at different time intervals i.e., day 0, 1, 3, 6, 9, 12, 15, 18 and 21. Tissue myeloperoxidase, superoxide anion and total calcium levels were determined after 21^st ^day to assess biochemical alterations. Histopathological evaluations were also performed. Hydroalcoholic extract of *Acorus calamus *(HAE-*AC*, 100 and 200 mg/kg, *p.o.*) and pregabalin (10 mg/kg, *p.o.*) were administered from the day of surgery for 14 days.

**Results:**

CCI of sciatic nerve significantly induced thermal, radiant, mechanical hyperalgesia and thermal, chemical, tactile allodynia, along with increase in the levels of superoxide anion, total calcium and myeloperoxidase activity. Moreover significant histological changes were also observed. HAE-*AC *attenuated CCI induced development of painful behavioural, biochemical and histological changes in a dose dependent manner similar to that of pregabalin serving as positive control.

**Conclusions:**

*Acorus calamus *prevented CCI induced neuropathy which may be attributed to its multiple actions including anti-oxidative, anti-inflammatory, neuroprotective and calcium inhibitory actions.

## Background

Pain after injury to the nervous system (neuropathic pain) is a major chronic pain condition that remains difficult to treat. Both peripheral and central mechanisms of neuropathic pain have been proposed by various researchers [1-4]. Neuropathic pain associated with peripheral nerve injury is characterized by the sensory abnormalities such as unpleasant abnormal sensation (dysesthesia), an increased response to painful stimuli (hyperalgesia), and pain in response to a stimulus that does not normally provoke pain (allodynia) [4]. Peripheral neuropathic pain is frequently observed in patients with long standing diabetes, cancer, AIDS, leprosy, cervical disc protrusion and foraminotomy and after surgery [5-9]. Chronic constriction injury of sciatic nerve induced painful neuropathy is a widely employed model for induction of neuropathic pain in experimental animals [10]. CCI induced neuropathy in experimental animals mimics Complex Regional Pain Syndrome - reflex sympathetic dystrophy (CRPS-RSD) in humans [11-13], which is common following fracture, total knee arthroplasty and stroke [14-17].

Conventional analgesics like non-steroidal anti-inflammatory drugs and opioids are in-effective clinically in attenuating neuropathic pain. Further, tricyclic anti-depressants (i.e., amitriptyline, nortriptyline and imipramine) and anti-convulsants (i.e., phenytoin, carbamazepine, gabapentin, lamotrigine and topiramate) have also been reported to produce anti-allodynic effects in neuropathy [18-22]. However, these drugs are reported to exhibit a wide spectrum of adverse effects which limit their full clinical exploitation in management of painful neuropathy [23-25]. Moreover, none of the medications assessed in randomized controlled studies conducted has been found effective in CRPS [26-28].

Preclinically, various studies have reported herbal medicine to produce the beneficial effect in the management of painful neuropathy i.e., *Aconiti tuber*, *Lindera angustifolia*, *Teucrium polium*, *Phyllanthus emblica*, *Vochysia divergens, Cannabis sativa*, *Nigella sativa*, *Ocimum sanctum *and *Ginkgo biloba *[29-33]. Therefore, there is an ample scope of new medicine from plant origin to combat the neuropathic pain conditions. Some recent clinical reports have also advocated beneficial effect of drugs from plant origin in neuropathic pain conditions [34-36].

*Acorus calamus *(family: *Araceae*), is an indigenous plant, traditionally, it is used as an ingredient of various cocktail preparations employed for the treatment and management of headache, migraine, body ache and severe inflammatory pain of the Unani, Ayurveda and local health care systems in Indian system of medicine. Fresh rhizome part extraction and/or decoction of *Acorus calamus *commonly used to relieve the muscle, joint, vascular and nerve injury associated severe inflammatory and neuropathic pain in south Indian population. Phytochemically it has reported the presence of glycosides, flavonoids, saponins, tannins, polyphenolic compounds, mucilage, volatile oil and bitter principles [37,38]. Experimental reports have indicated that, the rhizome part of the *Acorus calamus *plant has several medicinal properties, which is used in the treatment of insomnia, melancholia, neurosis, remittent fevers, delirium and hysteria [38]. The aqueous and hydroalcoholic extracts have been shown to express the hypolipidemic and neuropharmacological activities [39,40]. In our recent study, hydroalcoholic extract of *Acorus calamus *rhizome part has been shown to exert beneficial effect in tibial and sural nerve transection induced neuropathic pain in rats [41]. However usefulness of *Acorus calamus *in chronic constriction injury of sciatic nerve induced painful peripheral neuropathy remains to be explored. Therefore, the present study has been designed to investigate the ameliorative effect of *Acorus calamus *in chronic constriction injury of sciatic nerve induced neuropathic pain in rats. Pregabalin a selective Ca_v _2.2 (α_2_-δ subunit) channel antagonist served as positive control in this study.

## Methods

### Plant material

The fresh rhizome part of *Acorus calamus *were collected at Kodaikanal of Tamilnadu, India and authenticated through department of botany, American college, Madurai district, Tamilnadu. Plant sample has been kept in voucher specimen (PUP-218/2009-2010) at Punjabi University, Patiala for future reference. After authentication, fresh rhizome of *AC *were collected, cleaned thoroughly with distilled water and dried under shade. The shade dried rhizome was pulverized in a mechanical grinder to obtain coarse powder (sieve no.10/40).

### Extraction

The coarsely powdered plant material was subjected to extraction with mixture of ethanol:water (1:1, 50%) at room temperature. After completion of extraction, the solvent was completely removed by vacuum drying at low temperature (<50°C). The yield of hydroalcoholic extract was found to be 26.4% (w/w). The crude extracts were evaluated for absence of β-asarone by measuring the extinction at 253 and 303 nm by HPLC method. The extract was found to contain saponins, glycosides, flavonoids and tannins. This hydroalcoholic extract was used to explore its potential in chronic constriction injury of sciatic nerve induced painful neuropathy in rat.

### Chemicals

DTNB [5,5'-dithio, bis (2-nitro benzoic acid)], NBT (Nitro Blue Tetrazolium) (Sigma Aldrich, USA), BSA (Bovine Serum Albumin) (Sisco Research Laboratories Pvt. Ltd., Mumbai, India), hexadecyl trimethyl ammonium bromide (HETAB), O-dianisidine hydrochloride (S.D. Fine, Mumbai India), Folin-Ciocalteu's phenol reagent (Merck Ltd. Mumbai, India) were procured for the present study. All the chemicals used in the present study were of analytical grade.

### Experimental animals

Wistar rats (either sex), weighing between 200-230 g [procured from disease free small animal house, CCS-Haryana Agricultural University, Hisar] were used in the present study. The animals given free access to water and standard laboratory diet (Kisan Feeds Ltd., Mumbai, India) and were exposed to 12 h light and dark cycles. The experimental protocol was duly approved by the institutional animal ethics committee of Punjabi University, Patiala (Punjab), India, and the care of the animals was carried out as per guidelines of the committee for the purpose of control and supervision of experiments on animals, Ministry of environment and forest, Government of India (Reg. No.: 107/1999/CPCSEA).

### Induction of peripheral neuropathy by chronic constriction injury (CCI)

Peripheral neuropathy was induced in rats by chronic constriction injury as described method of Bennett and Xie [10] with slight modifications of Sommer and Schafers [42]. In brief, the rats were anesthetized with thiopental sodium (35 mg/kg i.p.). The hair of the rat's lower back in thigh region of left paw was shaved, and the skin was sterilized with povidone-iodine (Betadine™). The skin of the lateral surface of the left thigh was incised and a cut was made directly through the biceps femoris muscles to expose the sciatic nerve. Four ligatures (silk 4-0), were placed around the nerve proximal part of the trifurcation with a distance of 1 mm between each ligature. The ligatures were loosely tied until a short flick of the ipsilateral hind limb was observed. After performing nerve ligation, muscular and skin layer was immediately sutured with thread and topical antibiotic was applied at once. Nociceptive threshold was assessed before and after performing surgery on different days i.e. 0, 1, 3, 6, 9, 12, 15, 18, and 21^st ^day.

### Experimental protocol

Ten groups, each comprising of six Wistar rats, were employed in the present study.

Group I (Normal control): Rats were not subjected to any surgical procedure and were kept for 3 weeks. The behavioural tests were performed on the different days, i.e., day 0, 1, 3, 6, 9, 12, 15, 18 and 21^st^. Thereafter, all the animals were sacrificed subjected to biochemical analysis for the estimation of total protein, superoxide anion generation and total calcium in sciatic nerve tissue and myeloperoxidase activity in the surrounding muscular tissue sample.

Group II (Sham control): Rats were subjected to a surgical procedure (on day 0) to expose the sciatic nerve without any ligation. The behavioural tests were employed before performing the surgery and after 24 hr of surgery (i.e., day 1^st^) on different days as described in group I. The biochemical analysis was also done as mentioned in group I.

Group III (Chronic Constriction Injury, CCI): Rats were subjected to a surgical procedure to expose and four loose ligation of the sciatic nerve. The behavioural tests and the biochemical parameters were assessed as mentioned in group I.

Group IV (Vehicle in CCI): Carboxy methyl cellulose (CMC) (0.5% w/v, *p.o.*) was administered for 14 days (starting from day 1^st^) in rats subjected to CCI. The behavioural tests and the biochemical parameters were assessed as mentioned in group I.

Groups V (*AC *per se): Hydro-alcoholic extract of *AC *(200 mg/kg, *p.o.*) was administered for 14 days in normal rats, starting from the day 1^st^. The behavioural tests and the biochemical parameters were assessed as mentioned in group I.

Groups VI (Pregabalin per se): Pregabalin (10 mg/kg, *p.o.*) was administered for 14 days in normal rats, starting from the day 1^st^. The behavioural tests and the biochemical parameters were assessed as mentioned in group I.

Groups VII and VIII (Low and high dose of *AC *in CCI): Hydro-alcoholic extract of *AC *(100 and 200 mg/kg, *p.o.*) was administered for 14 days in rats subjected to CCI, starting from the day 1^st^. The behavioural tests and the biochemical parameters were assessed as mentioned in group I.

Groups IX (Pregabalin in CCI): Pregabalin (10 mg/kg, *p.o.*) was administered for 14 days in rats subjected to CCI, starting from the day 1^st^. The behavioural tests and the biochemical parameters were assessed as mentioned in group I.

Groups X (Spontaneous motor activity and motor co-ordination):

For testing effect on spontaneous motor activity and motor-co-ordination, photoactometer test and rota-rod tests respectively were employed. Each animal was first placed in photoactometer for 5 minutes followed by rota-rod test. After 1^st ^day measurements the animals were treated with hydro-alcoholic extract of *AC *(200 mg/kg, *p.o. *daily) for next 14 days. The test was repeated on 14^th ^day after 1 hour of drug administration.

### Behavioural studies

#### Heat hyperalgesic and allodynia test

Heat hyperalgesia and allodynia of the hind paw were assessed using Eddy's hot plate as described method of Eddy *et al. *[43], for assessing the reactivity to noxious and non-noxious thermal stimuli respectively. The rats were placed on the top of a controlled preheated (52.5 ± 0.5°C for hyperalgesia; 45 ± 0.5°C for allodynia) and maintained hot plate surface, allowing access to the left hind paw withdrawal response to degree of the nociceptive threshold. The cut-off times of 20 s for hyperalgesia and 30 s for allodynia were maintained.

#### Radiant heat hyperalgesic test

Radiant heat hyperalgesia of the left hind paw was assessed using the radiant heat lamp source as described method of Hargreaves *et al. *[44], for assessing the reactivity to noxious thermal stimuli. The intensity of the radiant heat stimulus was maintained at 25 ± 0.1°C. Response of left hind paw withdrawal threshold was noted. Cut-off time of 20 s was maintained.

#### Cold chemical allodynia test

Cold-allodynia of the hind paw was assessed using acetone drop method as described method of Choi *et al. *[45], with slight modification, for assessing the reactivity to non-noxious cold chemical stimuli. The rats were placed on the top of a wire mesh grid, allowing access to the hind paws. Acetone (0.1 ml) was sprayed on the plantar surface of left hind paw of rat. Cold chemical sensitive reaction with respect to either paw licking, shaking or rubbing the left hind paw was observed and recorded as paw lifting duration for 20 s test period.

#### Static mechanical hyperalgesic test

Mechanical (static) nociceptive threshold as an index of mechano-hyperalgesia were assessed by pressure stimulation method as described method of Randall and Selitto [46]. Briefly, nociceptive threshold, expressed in grams, as measured by applying increasing pressure to the left hind paw. Withdrawal of left hind paw or vocalization response was noted to assess the static mechanical nociceptive threshold. The cut-off pressure of 450 g was maintained.

#### Tactile mechanical hyperalgesic test

Mechanical (tactile) hyperalgesia was assessed by the pinprick test as described in the method of Erichsen and Blackburn-Munro [47]. The plantar surface of the injured left hind paw was touched with the point of the bent 18 gauge needle (at 90° angle) at intensity sufficient to produce a reflex withdrawal response in normal non-operated animals, but at an intensity which was insufficient to penetrate the skin in all other group. The duration of the paw withdrawal was recorded in seconds. A cut-off time of 20 s was maintained.

#### Mechanical allodynia test

Mechanical allodynia (non-noxious mechanical stimuli) was assessed as described by Chaplan *et al. *[48]. Briefly, calibrated nylon filaments [Von Frey Hair, total 20 filaments numbered 1.65, 2.36, 2.44, 2.83, 3.22, 3.61, 3.84, 4.08, 4.17, 4.31, 4.58, 4.74, 4.83, 5.07, 5.18, 5.48, 5.88, 6.10, 6.45, and 6.65], in terms of different bending forces (0.008, 0.02, 0.04, 0.07, 0.16, 0.4, 0.6, 1, 1.4, 2, 4, 6, 8, 10, 15, 26, 60, 100, 180, 300 g), were applied to the mid plantar surface of left hind paw. The filaments were applied ten times, starting with the softest and continuing in ascending order of stiffness. A brisk withdrawal of the left hind limb was considered a positive response. The criterion for the threshold value, in grams, was equal to the filament evoking a withdrawal threshold of the left hind paw 5 times out of 10 trials i.e., 50% response.

#### Motor co-ordination test

Motor co-ordination (grip muscle strength) was evaluated by a rota-rod device as described by Jones and Roberts [49] with slight modification of Muthuraman *et al. *[32]. Rats were placed for one minute on the rotating rod (25 rpm). The time taken for the falling from the roller, during one minute period was recorded.

#### Spontaneous locomotor (exploratory) activity test

Photoactometer test was employed to assess the effect of drug treatment on spontaneous motor (exploratory) activity. Each animal was observed for a period of 5 min in a square closed field arena (30 × 30 × 30 cm) equipped with 6 photocells in the outer wall. Interruptions of photocell beams (locomotor/exploratory action) were recorded by means of a 6 digits counter [50].

### Biochemical estimation

All the animals were sacrificed on 21^st ^day after surgery with chemical euthanasia (50 mg/kg, i.p., thiopental sodium). The injury site and distal portions of the sciatic nerve and the tissue beneath the sciatic nerve were isolated immediately. Further, the samples were kept in the humidity chamber (maintained at 85% relative humidity and 37°C temperature) to remove and maintain the moisture content of the collected samples. The sciatic nerve homogenate (10%, w/v) was prepared with 0.1 M Tris-HCl buffer (pH 7.4), and deionised water for total protein and total calcium estimation respectively. Superoxide anion measurement was carried out in sciatic nerve as described method of Wang *et al. *[51]. Further, surrounding muscular tissue was homogenates with phosphate buffer (pH 7.4) and employed for myeloperoxidase (MPO) estimation.

#### Estimation of total protein content

Protein concentration was estimated according to the method of Lowry *et al. *[52], Using Bovine serum albumin (BSA) as a standard. The absorbance was determined spectrophotometrically at 750 nm.

#### Estimation of superoxide anion generation

The sciatic nerve superoxide anion generation was estimated in terms of reduced nitroblue tetrazolium (NBT) as described in the method of Wang *et al. *[51]. Briefly, sciatic nerve homogenate react with NBT under certain chemical environment to form formazan as an index of superoxide anion generation. The absorbance of formazan was determined spectrophotometrically at 540 nm.

The quantity of NBT reduction = A x V/(T x Wt x ε x l),

Where A is the absorbance of blue formazan at 540 nm, V is the volume of the solution, T is the time period (90 minutes) during which rings were incubated with NBT, Wt is the blotted wet weight of the sciatic nerve, ε is the extinction coefficient of blue formazan (i.e., 0.72 l/mmol/mm), and l is the length of the light path. Results were reported as picomoles per minute per milligram wet weight of sciatic nerve.

#### Estimation of myeloperoxidase (MPO) activity

The myeloperoxidase activity was measured by a method described by Grisham *et al. *[53]. The absorbance was read spectrophotometrically at 460 nm. One unit of the myeloperoxidase activity is defined as that which would produce a change in absorbance of 1.0 unit/min at pH 7.0 and 25°C, calculated from the initial rate of reaction with peroxide (1 μM) as the substrate. The results were expressed as myeloperoxidase activity units per milligram of protein at one minute.

#### Estimation of total calcium

Total calcium levels were estimated in sciatic nerve according to the method of Severinghaus and Ferrebee [54] with slight modification of Muthuraman *et al. *[32,55]. Briefly, sciatic nerve homogenate was mixed with 1 ml of trichloroacetic acid (4%) in ice cold conditions and centrifuged at 2000 r.p.m. for 10 minutes. The clear supernatant was used for the estimation of total calcium ion by atomic emission spectroscopy at 556 nm.

### Histopathological evaluation

Samples of distal portion of sciatic nerve were stored in the fixative solution (10% formalin) and cut into 4 μm thickness. Staining was done by using hematoxylin and eosin as described by method of Sudoh *et al. *[56]. Nerve sections were analyzed qualitatively under light microscope (450 X) for axonal degeneration.

### Statistical analysis

All the results were expressed as mean ± standard error of means (S.E.M.). The data from the behavioural results were statistically analyzed by two-way analysis of variance followed by Bonferonni's posthoc-test using GraphPad Prism Version-5.0 software. The data from the biochemical results were statistically analyzed using one-way ANOVA followed by Tukey's multiple range tests. The *p-*value < 0.05 was considered to be statistically significant.

## Results

Administration of hydroalcoholic extract of *Acorus calamus *did not produce any significant effect on motor-coordination and spontaneous motor (locomotor or exploratory) activity of rats as tested on rota-rod and photoactometer respectively (data not shown).

### Effect on heat hyperalgesia and allodynia

**Figure 1:**
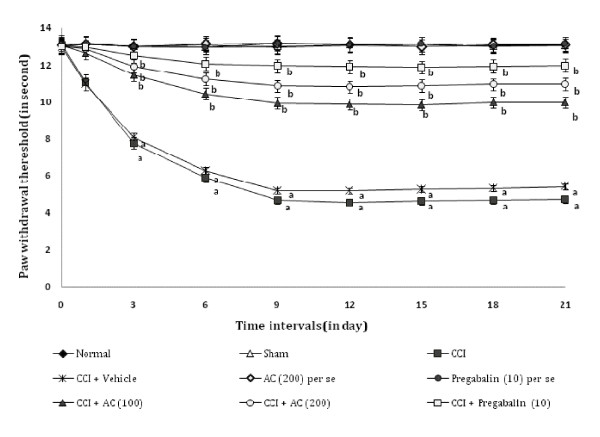
**Effect of *Acorus calamus *on heat hyperalgesia assessed by the noxious thermal sensation evoked ipsilateral hind paw withdrawal threshold**. Digits in parentheses indicate the dose of *Acorus calamus *and pregabalin in mg/kg. Data expressed as mean ± standard error of mean (SEM), each group consist of six (n = 6) Wistar rat. ^**a**^*p *< 0.05 vs sham control group. ^**b**^*p *< 0.05 vs CCI control group.

**Figure 2:**
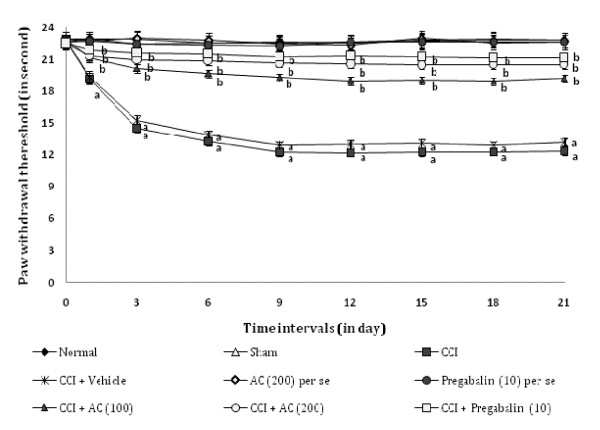
**Effect of *Acorus calamus *on heat allodynia assessed by the non-noxious thermal sensation evoked ipsilateral hind paw withdrawal threshold**. Digits in parentheses indicate the dose of *Acorus calamus *and pregabalin in mg/kg. Data expressed as mean ± standard error of mean (SEM), each group consist of six (n = 6) Wistar rat. ^**a**^*p *< 0.05 vs sham control group. ^**b**^*p *< 0.05 vs CCI control group.

Chronic constriction injury resulted in a significant development of thermal hyperalgesia (Figure 1) and allodynia (Figure 2) noted by decrease in left hind paw withdrawal threshold as compared to sham groups. Administration of hydroalcoholic extract of *AC *(100 and 200 mg/kg, *p.o.*) attenuated CCI induced decrease in the nociceptive threshold for thermal hyperalgesia [63.02% ± 0.041 and 74.54% ± 0.025 (inhibition ± SEM)] and allodynia [66.08% ± 0.019 and 79.98% ± 0.033 (inhibition ± SEM)] in a dose dependent manner. Treatment of pregabalin also produced similar effects [86.8% ± 0.026 for thermal hyperalgesia, and 86.84% ± 0.017 for allodynia (inhibition ± SEM)]. However, the vehicle administration did not modulate CCI induced thermal hyperalgesia and allodynia. Moreover, *AC *as well as pregabalin *per se *did not show any significant effect on above mentioned behaviour.

### Effect on radiant heat hyperalgesia

**Figure 3:**
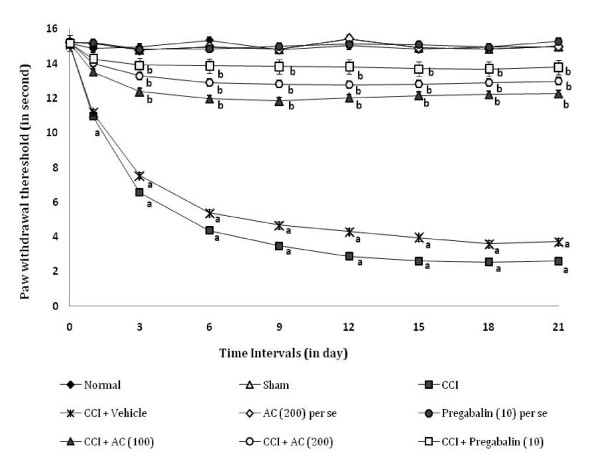
**Effect of *Acorus calamus *on radiant heat hyperalgesia assessed by the noxious thermal sensation evoked ipsilateral hind paw withdrawal threshold**. Digits in parentheses indicate the dose of *Acorus calamus *and pregabalin in mg/kg. Data expressed as mean ± standard error of mean (SEM), each group consist of six (n = 6) Wistar rat. ^**a**^*p *< 0.05 vs sham control group. ^**b**^*p *< 0.05 vs CCI control group.

CCI resulted in a significant development of radiant heat hyperalgesia (Figure 3) noted by decrease in left hind paw withdrawal threshold as compared to sham group. Administration of hydroalcoholic extract of *AC *(100 and 200 mg/kg, *p.o.*) attenuated CCI induced decrease in the nociceptive threshold for thermal hyperalgesia [75.6% ± 0.021 and 82.23% ± 0.028 (inhibition ± SEM)] in a dose dependent manner. Treatment of pregabalin also produced similar effects [89.88% ± 0.024 (inhibition ± SEM)]. However, the vehicle administration did not modulate CCI induced thermal hyperalgesia. Moreover, *AC *as well as pregabalin *per se *did not show any significant effect on above mentioned behaviour.

### Effect on cold chemical allodynia

**Figure 4:**
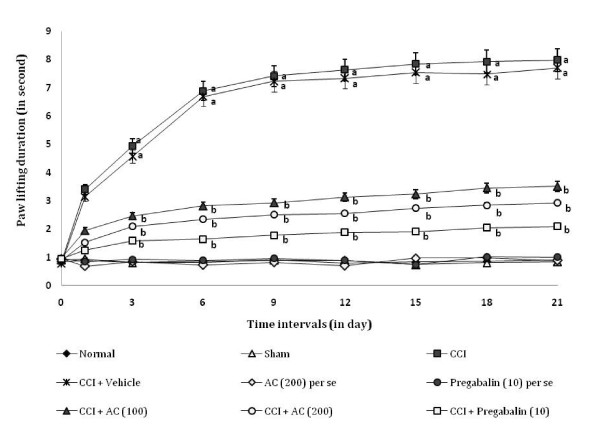
**Effect of *Acorus calamus *on cold chemical allodynia assessed by the non-noxious chemical stimuli evoked ipsilateral hind paw lifting duration**. Digits in parentheses indicate the dose of *Acorus calamus *and pregabalin in mg/kg. Data expressed as mean ± standard error of mean (SEM), each group consist of six (n = 6) Wistar rat. ^**a**^*p *< 0.05 vs sham control group. ^**b**^*p *< 0.05 vs CCI control group.

CCI resulted in a significant development of cold chemical allodynia as indicated by increase in the left hind paw lifting duration using acetone drop test method (Figure 4), as compared to sham group. Administration of *AC *(100 and 200 mg/kg) significantly attenuated CCI induced increase in paw lifting duration i.e. cold allodynia [65.67% ± 0.031 and 73.36% ± 0.016 (inhibition ± SEM)] in a dose dependent manner. Treatment of pregabalin also produced similar effects [84.6% ± 0.017 (inhibition ± SEM)]. However, the vehicle administration did not modulate CCI induced cold allodynia. Moreover, *AC *as well as pregabalin *per se *did not show any significant effect on above mentioned behaviour.

### Effect on static mechanical hyperalgesia

**Figure 5:**
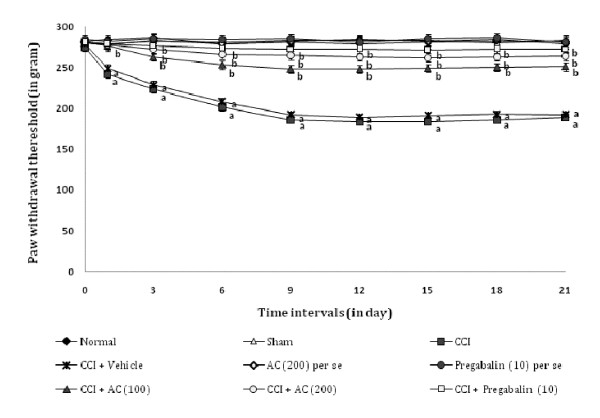
**Effect of *Acorus calamus *on static mechanical hyperalgesia assessed by the noxious mechanical stimuli evoked ipsilateral hind paw withdrawal threshold**. Digits in parentheses indicate the dose of *Acorus calamus *and pregabalin in mg/kg. Data expressed as mean ± standard error of mean (SEM), each group consist of six (n = 6) Wistar rat. ^**a**^*p *< 0.05 vs sham control group. ^**b**^*p *< 0.05 vs CCI control group.

CCI resulted in the development of static mechanical hyperalgesia as reflected by a significant increase in the left hind paw withdrawal threshold in the paw pressure test (Figure 5), as compared to sham group. Administration of hydroalcoholic extract of *AC *(100 and 200 mg/kg) attenuated CCI induced increase in the hind paw withdrawal threshold i.e. static mechanical hyperalgesia [66.42% ± 0.034 and 81.54% ± 0.025 (inhibition ± SEM)] in a dose dependent manner. Treatment of pregabalin also produced similar effects [90.9% ± 0.028 (inhibition ± SEM)]. However, the vehicle administration did not modulate CCI induced mechanical hyperalgesia. Moreover, *AC *as well as pregabalin *per se *did not show any significant effect on above mentioned behaviour.

### Effect on tactile mechanical hyperalgesia

**Figure 6:**
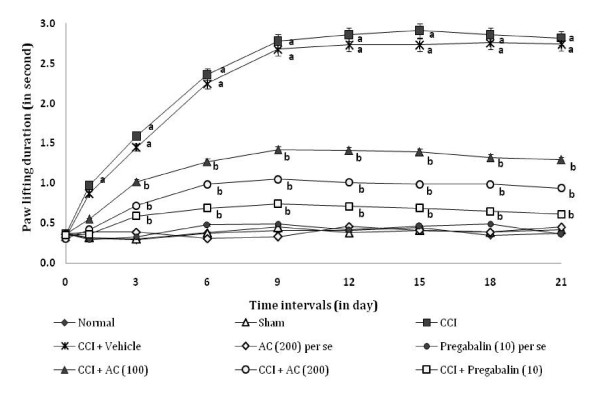
**Effect of *Acorus calamus *on tactile mechanical hyperalgesia assessed by the noxious mechanical stimuli evoked ipsilateral hind paw withdrawal duration**. Digits in parentheses indicate the dose of *Acorus calamus *and pregabalin in mg/kg. Data expressed as mean ± standard error of mean (SEM), each group consist of six (n = 6) Wistar rat. ^**a**^*p *< 0.05 vs sham control group. ^**b**^*p *< 0.05 vs CCI control group.

CCI resulted in the development of tactile mechanical hyperalgesia as reflected by a significant increase in the left hind paw lifting duration in the pinprick test (Figure 6), as compared to sham group. Administration of hydroalcoholic extract of *AC *(100 and 200 mg/kg) attenuated CCI induced increase in the hind paw lifting duration i.e. tactile mechanical hyperalgesia [59.5% ± 0.013 and 74.27% ± 0.016 (inhibition ± SEM)] in a dose dependent manner. Treatment of pregabalin also produced similar effects [87.78% ± 0.029 (inhibition ± SEM)]. However, the vehicle administration did not modulate CCI induced tactile mechanical hyperalgesia. Moreover, *AC *as well as pregabalin *per se *did not show any significant effect on above mentioned behaviour.

### Effect on mechanical allodynia

**Figure 7:**
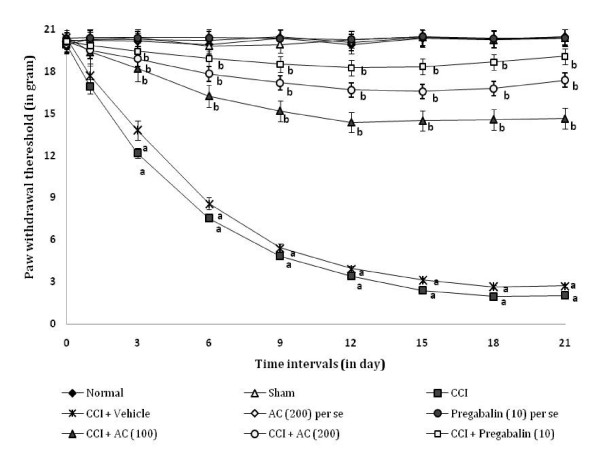
**Effect of *Acorus calamus *on mechanical allodynia assessed by the non-noxious mechanical stimuli evoked ipsilateral hind paw withdrawal threshold**. Digits in parentheses indicate the dose of *Acorus calamus *and pregabalin in mg/kg. Data expressed as mean ± standard error of mean (SEM), each group consist of six (n = 6) Wistar rat. ^**a**^*p *< 0.05 vs sham control group. ^**b**^*p *< 0.05 vs CCI control group.

CCI resulted in the development of tactile mechanical allodynia as reflected by a significant increase in the left hind paw withdrawal threshold in the Von Frey Hair test (Figure 7), as compared to sham group. Administration of hydroalcoholic extract of *AC *(100 and 200 mg/kg) attenuated CCI induced increase in the left hind paw lifting duration i.e. mechanical allodynia [67.99% ± 0.024 and 81.08% ± 0.026 (inhibition ± SEM)] in a dose dependent manner. Treatment of pregabalin also produced similar effects [90.53% ± 0.032 (inhibition ± SEM)]. However, the vehicle administration did not modulate CCI induced mechanical allodynia. Moreover, *AC *as well as pregabalin *per se *did not show any significant effect on above mentioned behaviour.

### Effect on tissue biomarker changes

CCI resulted in a significant increase in the levels of superoxide anion and total calcium in sciatic nerve and myeloperoxidase activity in surrounding tissue of sciatic nerve, as compared to sham group. Administration of the hydroalcoholic extract of *AC *(100 and 200 mg/kg *p.o.*) significantly attenuated CCI induced increase in the levels of superoxide anion [77.39% ± 1.18 and 86.46% ± 1.07 (inhibition ± SEM)], total calcium [75.16% ± 1.61 and 84.21% ± 1.34 (inhibition ± SEM)] and myeloperoxidase [76.62% ± 4.13 and 82.28% ± 3.36 (inhibition ± SEM)] levels, in a dose dependent manner. Treatment of pregabalin also produced similar effects on superoxide anion generation [98.39% ± 0.19 (inhibition ± SEM)], total calcium [98.31% ± 1.91(inhibition ± SEM)] and myeloperoxidase activity 98.87% ± 1.21]. However, the vehicle administration did not modulate CCI induced alteration in the superoxide anion generation and the total calcium levels. Moreover, as well as pregabalin *AC per se *did not show any significant effect on above mentioned biochemical levels (Table 1).

**Table 1:** Effect of *Acorus calamus *on tissue biomarker changes

Groups	Reduction of NBT (pmol/min/mg of protein)	Total Calcium (ppm/mg of protein)	MPO activity level (U/min/mg of protein)
Normal	3.38 ± 1.18	3.92 ± 1.12	10.12 ± 1.04
Sham	3.42 ± 1.45	3.35 ± 0.73	12.37 ± 1.83
CCI	21.56 ± 1.26 ^**a**^	33.43 ± 1.51 ^**a**^	165.24 ± 3.89 ^**a**^
CCI + vehicle	22.88 ±1.69 ^**a**^	37.36 ± 1.38 ^**a**^	159.63 ± 4.01 ^**a**^
*AC *(200) *per se*	3.40 ± 1.19	3.57 ± 0.64	11.94 ± 1.09
Pregabalin (10) *per se*	3.38 ± 1.23	3.25 ± 1.17	12.08 ± 0.92
CCI + *AC *(100)	7.49 ± 1.18 ^**b**^	11.26 ± 1.61 ^**b**^	47.36 ± 4.13 ^**b**^
CCI + *AC *(200)	5.84 ± 1.07 ^**b**^	8.59 ± 1.34 ^**b**^	38.65 ± 3.36 ^**b**^
CCI + Pregabalin (10)	3.27 ± 0.19 ^**b**^	4.43 ± 1.91 ^**b**^	13.14 ± 1.21 ^**b**^

Digits in parentheses indicate the dose of *Acorus calamus *and pregabalin in mg/kg.

Data expressed as mean ± standard error of mean (SEM), each group consist of six (n = 6) Wistar rat.

^**a**^*p *< 0.05 vs sham control group.

^**b**^*p *< 0.05 vs CCI control group.

### Effect on histopathological changes

**Figure 8:**
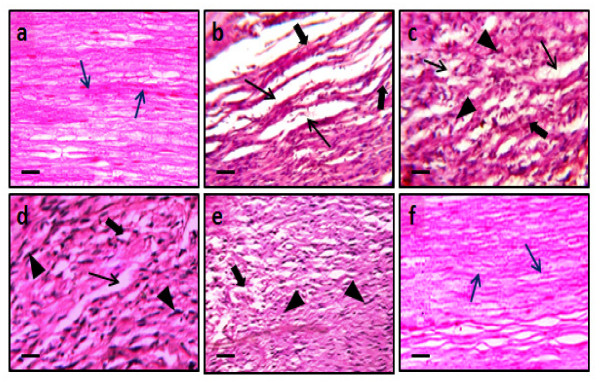
**Effect of *Acorus calamus *on chronic constriction injury induced histopathological changes**. Figures 8a-8f show transverse-section of sciatic nerve of sham, CCI, vehicle, *AC *(100 and 200 mg/kg) and pregabalin pretreated groups respectively. In figure 8a and 8f, blue arrow shows normal fiber arrangement. In figures 8b-8e, black arrows show fiber derangement (thin arrow), swelling (bold arrow) of nerve fiber and presence of activated satellite cells and Schwann cells (arrow head). In figures 8d-8f, show attenuation of CCI induced swelling of nerve fibers by AC (100 and 200 mg/kg) and pregabalin pretreatment groups respectively. Microscopic examinations were performed under 450 × light microcopy, scale bar 35 μm.

CCI resulted in significant histopathological changes assessed in transverses section of the sciatic nerve. In transverse section, nerve derangement, axonal swelling, increase in number of Schwann and satellite cells were also noted. Administration of the hydroalcoholic extract of *AC *(100 and 200 mg/kg *p.o.*) significantly attenuated CCI induced fiber derangement, swelling of nerve fiber and activation of neuroglial cell (satellite cells and Schwann cells) as marker of histopathological alterations (Figure 8). Microscopic examinations were performed under 450 × light microcopy, scale bar 35 μm.

## Discussion

In the present study, *Acorus calamus *attenuated sciatic nerve ligation i.e., chronic constriction injury induced behavioural [i.e., thermal, radiant, mechanical, chemical sensation (hyperalgesia and allodynia)], biochemical (superoxide anion, myeloperoxidase and total calcium) and histopathological (axonal degeneration) changes. However no significant effect on motor-coordination and spontaneous (locomotor or exploratory) motor activity was observed. The behavioural alterations started on 3^rd ^day after the chronic constriction injury of sciatic nerve and lasted throughout the experimental period. These observations are in line with our earlier findings [32,56] and reports from the other laboratories [57,22]. In response to an injury to a nerve, initial steps of inflammatory reactions, involve the release of pro-inflammatory mediators from the resident macrophages, Schwann cells and area adjacent to nerve lesion [58]. It has been documented that the sustained activation of peripheral nociceptors leads to the hypersensitivity of the primary afferent neurons and central sensitization of the dorsal horn neurons [4].

Neuropathic pain (including CCI of sciatic nerve) has been demonstrated to produce a rise in tissue total calcium levels [55,59]. Further, a key role of calcium accumulation has also been reported in formalin, post-traumatic, axotomy, CCI and vincristine-induced models of neuropathic pain [32,55,60]. Calcium ion accumulation has been documented to trigger the secondary messengers i.e., activation of calcium binding protein (calpain and calmodulin) and calcium dependent kinase and phosphatase action. It can alter the homeostasis function of nervous system and enhancement of auto-destruction including long term potentiation, long term depression and neuronal hyper-excitation [61]. Calcium-induced activation of calpains has been shown to be responsible for the axonal degeneration by alteration of stability of axonal cytoskeleton protein [62].

Several studies evidenced that free radical and calcium mediated oxidative stress and inflammation together play a major role in the pathogenesis of neurodegenerative diseases, such as amyotrophic lateral sclerosis, Alzheimer's disease, Parkinson's disease and neuropathic pain [55,63,64]. Moreover, reactive oxygen and nitrogen species have also been well documented to contribute in the pathophysiological changes in long standing diabetes, toxin, Freund's adjuvant induced inflammation, chronic constriction injury and axotomy of sciatic nerve and ischemia-reperfusion of femoral artery mediated neuropathic pain [2,32,55,64-66].

In the present study, *Acorus calamus *has been observed to attenuate CCI induced behavioural, biochemical as well as histopathological changes. *Acorus calamus *is reported to exert a battery of beneficial effects in various ailments viz; epilepsy, memory deficits, rheumatic pain, neuralgia [38,39,67]. Ethanolic extract of *AC *has been reported to exert anti-cellular, immunosuppression actions along with inhibition of nitric oxide, interleukin-2 and tumor necrosis factor-alpha productions and blocking of voltage activated calcium channel [68,69]. Further ethanolic extract of *AC *has also been demonstrated to possess potential anti-oxidative, anti-inflammatory as well as neuroprotective actions [70-72]. Experimentally, it has also been reported to decrease free radical generation via enhancement of anti-oxidant mechanisms such as increase in superoxide dismutase, catalase, reduced glutathione and glutathione peroxidase levels [73]. Additionally, it is shown to be a neuroprotective action against the hypoxic (ischemia) event and chemical (i.e., acrylamide) induced severe insult in nervous system [74,75,61]. On the basis of data in hand and with support from literature, therefore, it may be proposed that hydroalcoholic extract of *AC *rhizome produced ameliorative effect in CCI induced painful peripheral neuropathy which may be attributed to its multiple effects viz; anti-oxidative, anti-inflammatory, and neuroprotective actions manifested in the terms of alleviation of CCI induced behavioural (hyperalgesia and allodynia), biochemical (superoxide anions, total calcium and MPO activity) as well as histopathological changes. Our findings support the contention that generation of reactive oxygen species, calcium channel over-activation and inflammation may be a major culprit in the axonal degeneration and pathogenesis of neuropathic pain.

Pregabalin [(S)-3-(aminomethyl)-5-methylhexanoic acid or S-(+) - isomer of 3 - isobutyl γ-aminobutyric acid] is an anti-convulsant that successfully treats many neuropathic pain syndromes [76]. Pregabalin is an Selective Ca_v _2.2 (α2-δ subunit) channel antagonist. It has potential actions like predecessor gabapentin, it's a structural analogue (but not functional) of the gamma aminobutyric acid. Pregabalin has analgesic, anti-convulsant and anxiolytic activities [77]. Preclinical trials have demonstrated an anti-hyperalgesic and anti-allodynic effect of pregabalin in various animal models of neuropathic pain [76,78,79]. Data of our study also supports these reports.

Since pregabalin is well documented to exert its beneficial effect in neuropathic pain via inhibition of voltage gated calcium [Ca_V _2.2 (α2-δ subunit)] channels and *Acorus calamus *has also been shown to modulate calcium channel activity [68]. Therefore, it is proposed that in addition to its potential anti-oxidative, anti-inflammatory and neuroprotective actions, voltage activated calcium channel [Ca_V _2.2 (α2-δ subunit)] modulatory action of *Acorus calamus *may be an important factor in attenuating CCI induced peripheral neuropathic pain. Nevertheless further studies are needed to substantiate these findings.

## Conclusion

*Acorus calamus *attenuated the chronic constriction injury induced behavioural, biochemical and histopathological changes in rats. These effects may be attributed to its potential anti-oxidative, anti-inflammatory, neuroprotective and calcium inhibitory actions.

## Competing interests

The authors declare that they have no competing interests.

## Authors' contributions

AM participated in the experimental study and performed for the statistical analysis. NS participated in its design and helped to draft the manuscript. All authors read and approved the final manuscript for publication.

## Pre-publication history

The pre-publication history for this paper can be accessed here:

http://www.biomedcentral.com/1472-6882/11/24/prepub
